# Reliability of coronal curvature measurements on 3D ultrasound images for AIS

**DOI:** 10.1186/1748-7161-10-S1-O37

**Published:** 2015-01-19

**Authors:** Edmond Lou, Rui Zheng, Amanda CY Chan, Douglas L Hill, Marc J Moreau, Douglas M Hedden, James K Mahood, Sarah Southon

**Affiliations:** 1 University of Alberta, Alberta. Canada; 2 Alberta Health Services, Alberta, Canada

## Objective

Ionizing radiation exposure is a concern for children with scoliosis. A pilot study demonstrated that a proxy Cobb angle can be obtained from spinal ultrasound (US) images. The objective was to evaluate the reliability and comparison to radiographic Cobb angle of the coronal curvatures measurements from the US images on AIS patients.

## Methods

Forty participants (35F, 5M; mean age 14.0±2.0 years) who (1) were diagnosed with AIS, (2) had no prior spine surgical treatment, (3) had radiographs on the study day that were not in-brace, and (4) had the major Cobb angle less than 45°, were recruited consecutively from the local scoliosis clinics. All US images and radiographs were acquired in standing positions within an hour of each other. Three raters, who were blinded to the clinical information, measured the Cobb angles from the US and x-ray images at least 3 days apart. The raters repeated these measurements at least 1 week apart to minimize memory bias. The mean absolute deviation (MAD) and the standard deviation (SD) between the two measurements methods were used to estimate the reliability of the US measurement. The intra- and inter-rater reliabilities were assessed by calculating the interclass correlation coefficients and standard errors of measurement (SEM).

## Results

78 curves (23.3±6.8° ; range:12-45°) including 44 major curves (25.2±7.3°)were recognized from the radiographs. 63 curves including 42 major curves were detected on the US images. Among the 3 raters, the MAD±SD between the US and radiography measurements were 3.8±2.7°, 4.3±3.1°, and 3.4±2.8°, respectively. The ICC(2,1) values of the intra- and inter-rater reliability of the US measurements ranged from 0.85-0.94, and 0.80-0.87, respectively. The correlation coefficient (R) between the two methods ranged from 0.71-0.76 for major curves and 0.73-0.79 for all curves. The SEM of the major curves and overall were 3.4° and 3.8°, respectively.

## Conclusions

Although only 80% of the curves could be recognized from the US images, 95% of the major curves were detected on US images. Most of the curves missed using ultrasound were non-treated curves (non-structural), many of which were in the upper thoracic region. The mean differences between the two measurement methods were within the clinically accepted error of 5°. The high ICC values indicate that the US method is reliable. Further work will explore sensitivity to change in US compared to radiographic Cobb. Use of US for scoliosis monitoring may reduce ionizing exposure to children with scoliosis by replacing some radiographs.

